# A Novel Non-Enzymatic Electrochemical Hydrogen Peroxide Sensor Based on a Metal-Organic Framework/Carbon Nanofiber Composite

**DOI:** 10.3390/molecules23102552

**Published:** 2018-10-06

**Authors:** Yijun Fu, Jiamu Dai, Yan Ge, Yu Zhang, Huizhen Ke, Wei Zhang

**Affiliations:** 1 College of Textile and Clothing, Nantong University, Nantong 226019, China; fuyj@ntu.edu.cn (Y.F.); jmdai@ntu.edu.cn (J.D.); ntdxgeyan@126.com (Y.G.); z.yu@ntu.edu.cn (Y.Z.); 2 National & Local Joint Engineering Research Center of Technical Fiber Composites for Safety and Protection, Nantong 226019, China; 3 Fujian Key Laboratory of Novel Functional Textile Fibers and Materials, Minjiang University, Fuzhou 350108, China

**Keywords:** metal-organic framework, ZIF-67, non-enzymatic sensor, carbon nanofiber, hydrogen peroxide

## Abstract

A co-based porous metal-organic framework (MOF) of zeolitic imidazolate framework-67 (ZIF-67) and carbon nanofibers (CNFs) was utilized to prepare a ZIF-67/CNFs composite via a one-pot synthesis method. Scanning electron microscopy (SEM), energy dispersive X-ray spectroscopy (EDX), and X-ray diffraction (XRD) were employed to investigate the morphology, structure, and composition of the resulting composite. A novel high-performance non-enzymatic electrochemical sensor was constructed based on the ZIF-67/CNFs composite. The ZIF-67/CNFs based sensor exhibited enhanced electrocatalytic activity towards H_2_O_2_ compared to a pure ZIF-67-based sensor, due to the synergistic effects of ZIF-67 and CNFs. Meanwhile, chronoamperometry was utilized to explore the detection performance of the sensor. Results showed the sensor displayed high-efficiency electrocatalysis towards H_2_O_2_ with a detection limit of 0.62 μM (S/N = 3), a sensitivity of 323 µA mM^−1^ cm^−2^, a linear range from 0.0025 to 0.19 mM, as well as satisfactory selectivity and long-term stability. Furthermore, the sensor demonstrated its application potential in the detection of H_2_O_2_ in food.

## 1. Introduction

Hydrogen peroxide (H_2_O_2_), as an important industrial chemical, has been widely applied in various fields, including food production, medicine manufacture, clinic treatment, etc [1,2,3]. The detection of H_2_O_2_ in food is of great importance because it participates in various cellular metabolism pathways of the human body and an abnormal level of H_2_O_2_ may lead to severe diseases such as cardiovascular disorders, Alzheimer’s disease and even cancer [4,5,6]. Therefore, the development of fast and accurate methods for H_2_O_2_ detection has become a research hotspot. There are various kinds of methods for monitoring H_2_O_2_, such as chemiluminescence, titrimetry, electrochemistry, spectrometry and high performance liquid chromatography [7,8,9,10,11]. Among these methods, the electrochemical technique using natural enzymes is becoming increasingly popular due to its intrinsic advantages including simple operation, low cost, good selectivity, and high sensitivity [12,13]. Nevertheless, the performance of enzyme-based electrochemical sensors is mainly dependent on the enzyme activity, which is highly vulnerable to environmental factors like pH, temperature, etc [14]. Thus, novel non-biological materials have been exploited instead of enzymes in electrochemical sensors for H_2_O_2_ detection [10,12,13,15].

**Scheme 1:**
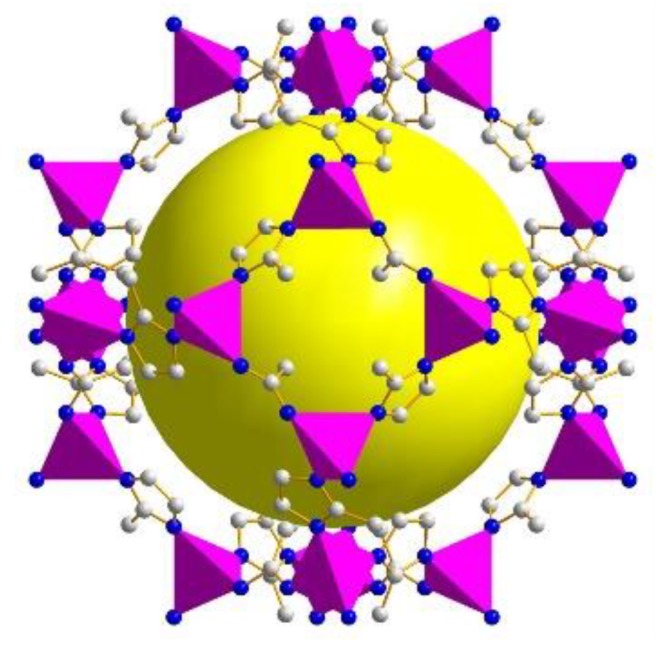
Crystal structure of ZIF-67.

Metal organic frameworks (MOFs), which are novel three dimensional organic-inorganic hybrids self-assembled by metal ions and organic ligands, have seen explosive growth in a variety of application fields during the past decades [16]. Generally, MOFs possess various topologies, and their morphology or constitution can be adjusted through the appropriate selection of metal ions, ligands, or synthesis methods [17]. These characteristics enable MOFs to be applied in gas storage, drug delivery, separation, catalysis, sensing, etc [18,19,20,21,22]. In this work, a Co-based porous MOF [Co(mIM)_2_]_n_ (denoted as ZIF-67, mIM = 2-methylimidazole) was selected as the modification material of an electrochemical sensor due to the favorable electrocatalytic property of Co^2+^ inside ZIF-67 towards H_2_O_2_ [23]. The crystal structure of ZIF-67 is shown in Scheme 1. The largest cage is shown with CoN_4_ in pink polyhedra, and the links in ball-and-stick presentation. The yellow ball indicates space in the cage. H atoms are omitted for clarity (C, gray; N, blue) [24].

Though the ZIF-67 exhibits favorable electrocatalytic ability towards H_2_O_2_, its poor conductivity restricts its application in electrochemical H_2_O_2_ sensors. To solve this problem, some researchers synthesized MOFs/carbon nanomaterials composites, e.g., MOF/reduced graphene oxide (MOF/rGO) composite, to improve the conductivity of MOF, and it was successfully applied in the high-efficiency monitoring of H_2_O_2_ [16,17,25]. Among all carbon materials, carbon nanofibers (CNFs) have attracted a lot of attention because they are easily synthesized and possess many more edge sites compared with carbon nanotubes (CNTs). It is widely accepted that CNFs exhibit outstanding conductivity, high porosity, huge specific surface, as well as excellent mechanical strength. Therefore, CNFs are good candidate for the development of electrochemical sensors or biosensors [26].

In this work, ZIF-67 and CNFs were jointly selected to synthesize a MOF/CNFs composite, namely ZIF-67/CNFs, which was further employed to construct a novel non-enzymatic electrochemical sensor. Afterwards, a series of electrochemical characterizations were carried out to investigate the electrochemical properties of ZIF-67/CNFs composite. In addition, the selectivity, stability, and practical application of ZIF-67/CNFs based electrochemical sensor were also studied to evaluate its potential application in H_2_O_2_ detection.

## 2. Results and Discussions

### 2.1. Morphology, Chemical Component, and EIS Analysis

**Figure 1:**
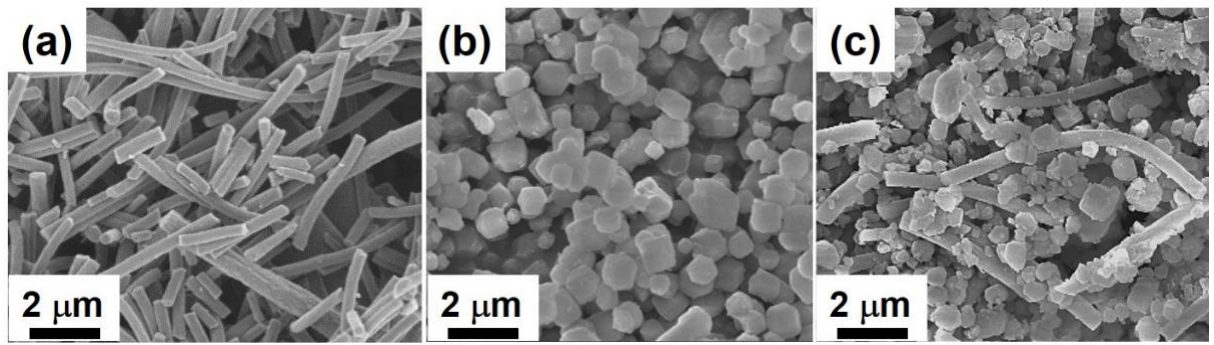
SEM images of (**a**) CNFs; (**b**) ZIF-67 nanocrystals; and (**c**) ZIF-67/CNFs composite.

Figure 1 shows the SEM images of CNFs, ZIF-67 nanocrystals, and ZIF-67/CNFs composite. Since CNFs were ground before the synthesis procedure, numerous short fibers are randomly distributed in Figure 1a. It can be observed that the surfaces of fibers were smooth, with no impurities on them. The diameter of the CNFs ranged from 213 nm to 524 nm, with an average diameter of 286 nm. It can be seen from Figure 1b that the ZIF-67 exhibited a dispersed rhombic dodecahedral nanocrystal morphology, which was in accordance with another report [27]. Moreover, the rhombic facet of ZIF-67 was well-defined and its edge was clearly visible, indicating the perfect crystalline structure of the synthesized ZIF-67 nanocrystals. The size of ZIF-67 nanocrystals was from 317 nm to 1.2 μm, with a mean particle size of 706 nm. Figure 1c presents the morphology of the ZIF-67/CNFs composite. It can be clearly seen that the ZIF-67 nanocrystals and CNFs were evenly mixed together, with some ZIF-67 nanocrystals attached on the surfaces of CNFs. This may be ascribed to the attractive forces between CNFs and ZIF-67 nanocrystals. Herein, the CNFs play the role of a “molecular wire” which could accelerate the electron transfer rate between ZIF-67 and electrode surface, leading to an improved electrocatalytic activity of ZIF-67/CNFs composite.

**Figure 2:**
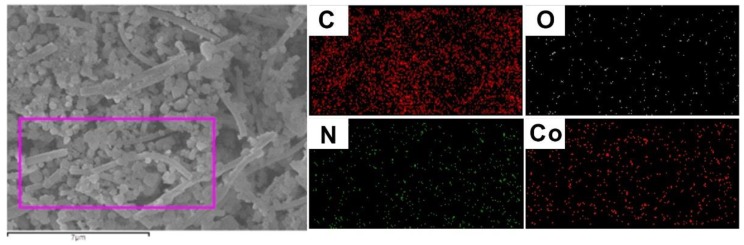
Element mapping images of the ZIF-67/CNFs composite.

Energy-dispersive spectroscopic (EDS) mapping was conducted to investigate the distribution of ZIF-67 nanocrystals in the ZIF-67/CNFs composite. As illustrated in Figure 2, it was apparent that the elements of C, O, N and Co were uniformly and densely dispersed in the ZIF-67/CNFs composite. Meanwhile, the existence of N and Co elements demonstrated that the ZIF-67 nanocrystals were successfully synthesized and introduced in the composite. In addition, considering the N and Co elements did not exist in CNFs, and these two elements were evenly dispersed in the EDS mapping picture, this suggested that the ZIF-67 nanocrystals were equally distributed in the ZIF-67/CNFs composite rather than aggregated.

**Figure 3:**
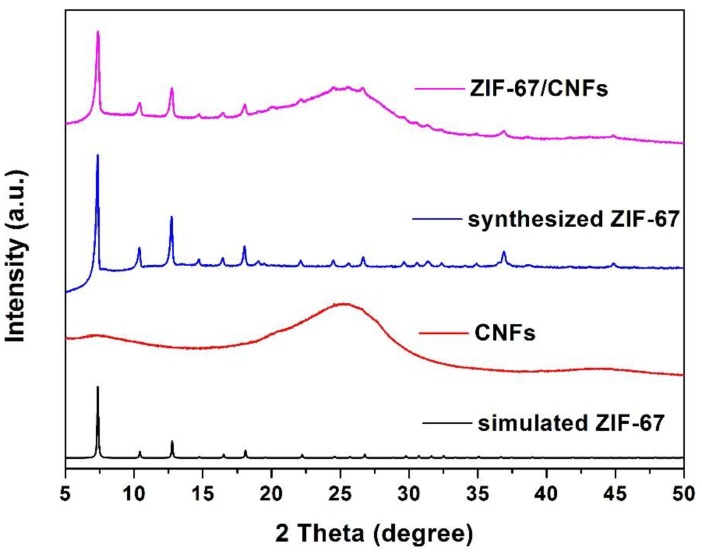
XRD patterns of simulated ZIF-67, CNFs, synthesized ZIF-67, and ZIF-67/CNFs composite.

X-ray diffraction (XRD) characterization was further employed to study the chemical components of ZIF-67/CNFs. Figure 3 shows the XRD patterns of CNFs, synthesized ZIF-67, and ZIF-67/CNFs composite. It can be clearly seen from Figure 3 that the CNFs displayed an apparent and wide diffraction peak at around 25.6°, which corresponded to the (002) crystalline plane of carbon material [28]. For the synthesized pure ZIF-67, it showed a pattern coincident with the simulated ZIF-67 one. Sharp peaks were clearly observed at the diffraction peaks of 7.3°, 12.4°, and 17.7°, which were assigned to the (110), (211), and (222) planes of ZIF-67 crystals, respectively [29]. Notably, the XRD pattern of ZIF-67/CNFs composite displayed the diffraction peaks of both CNFs and ZIF-67, indicating the successful synthesis of ZIF-67/CNFs composite.

**Figure 4:**
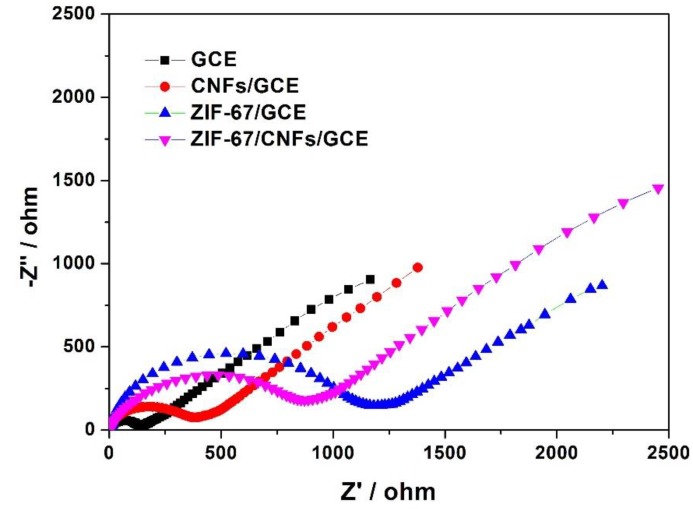
Nyquist plots for bare GCE, CNFs/GCE, ZIF-67/GCE and ZIF-67/CNFs/GCE in a solution of 0.1 M KCl containing 5 mM Fe(CN)_6_^3−/4−^ with the potential of 0.2 V, the frequency range from 0.01 Hz to 100,000 Hz and signal amplitude of 5 mV.

Electrochemical impedance spectroscopy (EIS) is an efficient method to explore the interface resistance of a modified electrode. In order to evaluate the potential application of ZIF-67/CNFs as an efficient electrocatalyst, it is essential to investigate the electron transfer resistance (Ret) of the ZIF-67/CNFs modified electrode. Figure 4 compares the Nyquist plots of different modified electrodes. It is well known that the semicircle diameter of the Nyquist plots corresponds to the value of Ret, and the Ret controls the electron transfer kinetics of the redox electrochemical probe at the electrode interface. As shown in Figure 4, the Ret value of bare GCE was 149 Ω, while the Ret value of ZIF-67/GCE was as high as 1206 Ω. However, the Ret value of ZIF-67/CNFs/GCE decreased to 876 Ω after the addition of CNFs. This indicates that the involvement of CNFs could significantly reduce the interface resistance of the modified electrode, leading to an improved conductivity.

### 2.2. Electrocatalytic Activity for H_2_O_2_ Reduction

**Figure 5:**
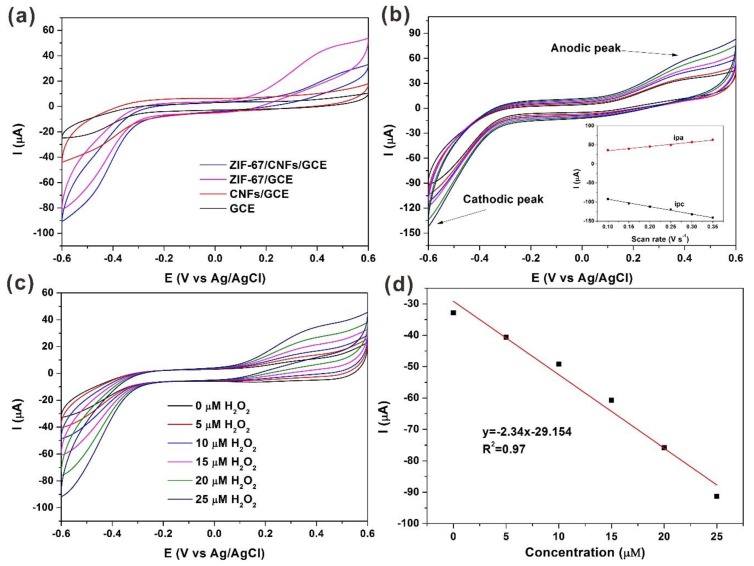
(**a**) CVs of different electrodes in 0.1 M NaOH solution containing 25 μM H_2_O_2_; (**b**) CVs of the ZIF-67/CNFs/GCE in 0.1 M NaOH solution at scan rates of 100, 150, 200, 250, 300, 350 mV s^−1^ (from inner to outer), respectively, inset: plots of the corresponding anodic and cathodic peak currents vs. scan rate; (**c**) CVs of the ZIF-67/CNFs/GCE in 0.1 M NaOH solution with the presence of varied H_2_O_2_ concentrations: 0, 5, 10, 15, 20, 25 μM (scan rate: 100 mV s^−1^); (**d**) the corresponding calibration curve.

Figure 5a shows the cyclic voltammograms (CVs) of bare GCE, CNFs/GCE, ZIF-67/GCE, and ZIF-67/CNFs/GCE in 0.1 M NaOH solution containing 25 μM H_2_O_2_. The peaks at −0.6 V were ascribed to the reduction reaction of H_2_O_2_. Apparently, CNFs/GCE displayed higher reduction peak current (44.8 μA) than bare GCE (24.3 μA), indicating the good electrocatalytic activity of CNFs. As for ZIF-67/GCE, it showed a much larger reduction peak current (81.5 μA) than that of CNFs/GCE, demonstrating the better electrocatalytic activity of ZIF-67 than CNFs. Interestingly, the reduction peak current of ZIF-67/CNFs/GCE was increased again by 9.1 μA in contrast with that of ZIF-67/GCE. The reason of this phenomenon can be ascribed as following: on one hand, the conductivity of the ZIF-67/CNFs composite was enhanced by CNFs; on the other hand, ZIF-67 and CNFs performed a synergistic catalysis role in the whole electrochemical reaction.

Figure 5b displays CVs of the ZIF-67/CNFs/GCE in 0.1 M NaOH solution at different scan rates. As can be seen from the insert of Figure 5b, the anodic and cathodic peak currents increased linearly as the scan rate increased from 100 to 350 mV s^−1^, suggesting a surface controlled electrochemical redox process, which indicated that the ZIF-67/CNFs composites were well immobilized on the electrode surface.

Figure 5c shows CVs of the ZIF-67/CNFs/GCE in 0.1 M NaOH solution with the presence of 0, 5, 10, 15, 20, and 25 μM of H_2_O_2_. As observed, the cathodic peak current values increased linearly with the concentration of added H_2_O_2_. Moreover, the calibration curve (shown in Figure 5d) also demonstrated the favorable linear relation between cathodic peak current value and H_2_O_2_ concentration. This result revealed that the ZIF-67/CNFs/GCE could find potential application as an electrochemical sensor.

**Scheme 2:**
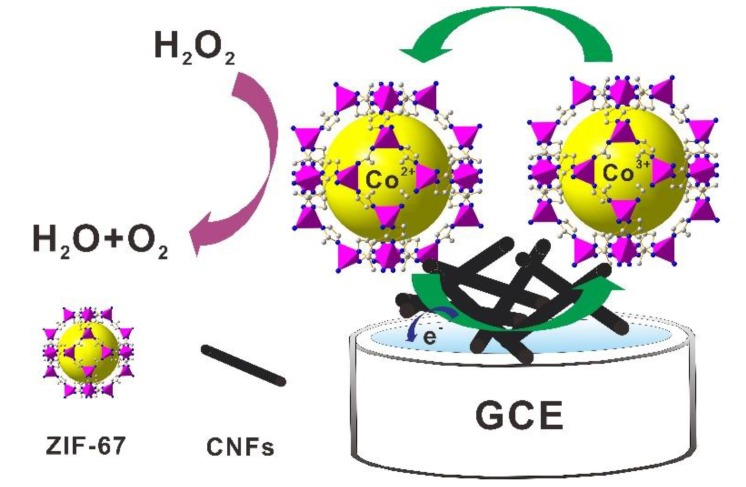
Schematic illustration of the reduction of H_2_O_2_ by ZIF-67/CNFs/GCE in NaOH solution.

The possible reaction mechanism is illustrated in Scheme 2 and the reaction path for ZIF-67 catalyst towards H_2_O_2_ could be expressed as following equations:

ZIF-67-Co^2+^ + H_2_O_2_ → ZIF-67-Co^3+^ + H_2_O + O_2_ (1)

ZIF-67-Co^3+^ + e^−^ → ZIF-67-Co^2+^ (2)

At the beginning of electrochemical reaction, H_2_O_2_ was adsorbed in the pores of ZIF-67-Co^2+^. As the reaction continued, the absorbed H_2_O_2_ was reduced into H_2_O and O_2_, along with the oxidization of ZIF-67-Co^2+^ into ZIF-67-Co^3+^. Last, ZIF-67-Co^3+^ achieved an electron from electrode to regenerate ZIF-67-Co^2+^. The CNFs existing in the composite membrane on electrode surface not only accelerated the electron transfer rate, but also worked together with ZIF-67 to achieve the synergistic catalysis, resulting in an amplified electrochemical signal.

### 2.3. Amperometric Response to H_2_O_2_

**Figure 6:**
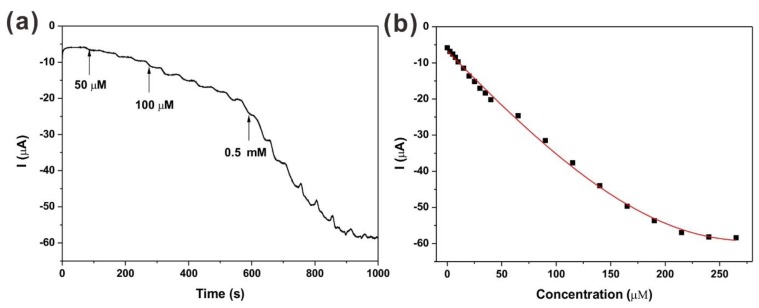
(**a**) Chronoamperometric responses of ZIF-67/CNFs/GCE on successive addition of different volume of H_2_O_2_ solutions into 0.1 M NaOH solution, applied potential: −0.2 V; (**b**) The calibration curves with nonlinear fitting.

The electroanalytical properties of ZIF-67/CNFs/GCE toward H_2_O_2_ were evaluated under −0.2 V. Figure 6a displays the chronoamperometric responses of ZIF-67/CNFs/GCE on successive addition of different volume of H_2_O_2_ solutions into 0.1 M NaOH solution. It can be seen from Figure 6a, with the successive addition of H_2_O_2_, the steady-state current values gradually increased. As shown in Figure 6b, the response current of the ZIF-67/CNFs/GCE showed a linear dependence on H_2_O_2_ concentration in the range from 2.5 µM to 190 µM with the linearity regression equation of Δ*i*(µA) = 9.244 + 0.217*c* (µM) (*r*^2^ = 0.986), a sensitivity of 323 µA mM^−1^ cm^−2^, and a limit of detection (LOD) of 0.62 µM (S/N = 3). Besides, non-linear fitting was also applied to show that when the concentration of H_2_O_2_ was higher than 190 µM, the response current tended to be stable, suggesting a detection limit of the sensor.

The sensing performance of the ZIF-67/CNFs/GCE was next compared with other reported non-enzymatic H_2_O_2_ sensors, and the results are summarized in Table 1. The prepared H_2_O_2_ sensor showed low detection limit, wide linear range, and high sensitivity, which are mainly attributed to excellent electrocatalytic activity of the ZIF-67/CNFs composite.

### 2.4. Reproducibility, Repeatability and Stability of the ZIF-67/CNFs/GCE

The ZIF-67/CNFs/GCE showed satisfactory reproducibility, repeatability, and stability. Five electrochemical sensors were independently prepared under the same conditions, and the relative standard deviation (RSD) of the five modified electrodes was 2.7%, indicating the electrochemical had good reproducibility.

**Figure 7:**
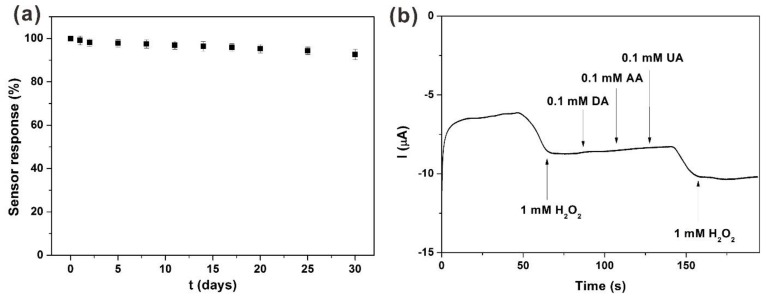
(**a**) Storage stability of the ZIF-67/CNFs/GCE in 0.1 M NaOH solution; (**b**) Chronoamperometric responses of ZIF-67/CNFs/GCE in 0.1 M NaOH solution at an applied potential of −0.2 V with 1mM H_2_O_2_ + 0.1 mM DA + 0.1 mM AA + 0.1 mM UA.

The RSD of response currents of 10 successive measurements of the electrochemical sensor towards H_2_O_2_ was within 2.0%, indicating satisfactory repeatability of the electrochemical sensor. Figure 7a shows the storage stability of the ZIF-67/CNFs/GCE in 0.1 M NaOH solution. After a week of storage in 0.1 M NaOH solution, the current response of the electrochemical sensor almost kept stable. The current response still retained 92.6% of the initial value through 30 days of storage, demonstrating that the electrochemical sensor based on ZIF-67/CNFs/GCE exhibited good stability.

### 2.5. Anti-Interference and Real Sample Analysis

In addition, the ZIF-67/CNFs/GCE sensor also displayed good anti-interference performance. As shown in Figure 7b, when adding 1 mM H_2_O_2_, the response current apparently changed, while it was barely affected by adding 0.1 mM DA, 0.1 mM AA, and 1.0 mM UA. The favourable anti-interference performance can be attributed to the negative operating potential (−0.2 V) and the protection role of Nafion layer. Thus, the ZIF-67/CNFs/GCE sensor showed a good selectivity to H_2_O_2_. To demonstrate the practical application of the sensor, recovery experiments of H_2_O_2_ detection was carried out in milk samples, which were bought from a local supermarket, and the results are shown in Table 2. Five parallel experiments were conducted to study the recovery property of H_2_O_2_ in milk samples. It can be clearly observed that the recovery were all approach to 100%, and the relative standard deviation (RSD) was only 1.5%. All these data suggested that the H_2_O_2_ sensor based on ZIF-67/CNFs/GCE could be applied in analysis of H_2_O_2_ existing in beverage and food.

## 3. Materials and Methods

### 3.1. Chemicals and Reagents

Polyacrylonitrile (PAN, average molecular weight = 82,000) powder, *N*,*N*-dimethylformamide (DMF), methanol, 2-methylimidazole (C_4_H_6_N_2_, mIM), cobalt nitrate hexahydrate (Co(NO_3_)_2_·6H_2_O), potassium ferricyanide (K_3_[Fe(CN)_6_]), potassium hexacyanoferrate (K_4_[Fe(CN)_6_]·3H_2_O), NaOH, hydrogen peroxide (H_2_O_2_), dopamine (DA), ascorbic acid (AA), and urea (UA) were all purchased from the Sinopharm Group Chemical Reagent Co., Ltd. (Shanghai, China). All chemicals were of analytical grade and used without further purification. Nafion (5% *w*/*w*) was obtained from E. I. Du Pont Company (Wilmington, NC, USA). Besides, 0.1 M NaOH solution was used as a supporting electrolyte. All aqueous solutions were prepared with deionized water (DIW).

### 3.2. Apparatus

The morphology, elemental analysis, and chemical components of ZIF-67, CNFs, and ZIF-67/CNFs composite were respectively characterized by a field emission scanning electron microscope (FE-SEM, S4800, Hitachi, Tokyo, Japan), an energy dispersive X-ray spectroscopy (EDX, NORAN SYSTEM 7, Thermo Scientific, Carlsbad, CA, USA), and a Powder D8 Advance X-ray diffraction (XRD, AXS D8, Bruker, Coventry, UK). Electrochemical experiments were carried out using a CHI 660E electrochemical workstation (CH Instruments, Shanghai, China) at room temperature. The electrochemical measurements were implemented by a three-electrode cell with a GCE (4.0 mm in diameter, 12 mm^2^ in area, purchased from Gaoss Union Technology Co., Ltd., Wuhan, China), a platinum wire auxiliary electrode and an Ag/AgCl reference electrode (saturated KCl).

### 3.3. Preparation of Carbon Nanofibers

The carbon nanofibers (CNFs) were prepared by the following method. Firstly, 12 wt % PAN powder, as precursor, was dissolved in DMF solution with 8 h of magnetic stirring to prepare the electrospinning solution. After that, the obtained solution was poured in a syringe for electrospinning with a flow rate of 1 mL/h, a voltage of 16 kV, and a working distance of 20 cm, and respectively. Lastly, the final CNFs were prepared by carbonizing the PAN nanofibers through a high temperature furnace. The whole procedure was carried out in Ar atmosphere and can be summarized as follows: (1) heating up to 280 ℃ with a heating rate of 2 ℃ min^−1^, maintaining this temperature for 1 h to complete the pre oxidation treatment of nanofibers; (2) heating up to 900 ℃ at the rate of 5 ℃ min^−1^, keeping this temperature for 2 h to carbonize the nanofibers. The obtained carbon nanofibrous membrane was further ground to powder (short carbon nanofibers) for the following experiments.

### 3.4. Synthesis of ZIF-67/CNFs Composite

In this study, Co(NO_3_)_2_·6H_2_O and mIM were used as precursors to produce ZIF-67 nanocrystals. First of all, the CNFs and Co(NO_3_)_2_·6H_2_O (with the mass ratio of 1:1) were dispersed in 40 mL of methanol with slight stirring for 1 h at 45 ℃. After that, above solution was added into 40 mL of 2 mol L^−1^ mIM methanol solution. The solution gradually became purple and was incubated for 24 h at room temperature. The obtained products were gathered, washed for several times with ethanol and collected by centrifugation. For comparison, pure ZIF-67 nanocrystals were prepared by similar procedure except from the addition of CNFs.

### 3.5. Preparation of Electrochemical Sensors

Considering the optimal response and stability of modified electrode, among control-experiments, the concentration and mass ratio of Nafion and ZIF-67/CNFs were optimized. Ultimately, the electrochemical sensor was prepared by the mixture containing 1 wt % Nafion, and 1 mg mL^−1^ ZIF-67/CNFs.

The preparation process of the ZIF-67/CNFs modified GCE (ZIF-67/CNFs/GCE) is as follows: Firstly, 1 mg ZIF-67/CNFs composites were added into 1 mL DIW to prepare ZIF-67/CNFs suspension under continuous stirring. Afterward, a mixture containing ZIF-67/CNFs suspension and a certain volume of Nafion (5 wt %) were stirred for 1 h. Lastly, the ZIF-67/CNFs/GCE was prepared by dropping 10 µL of the mixture on the surface of processed GCE, which was polished by alumina followed by rinsing and ultrasonicating with DIW.

For comparison experiments, CNFs-modified GCE (CNFs/GCE) and ZIF-67 modified GCE (ZIF-67/GCE) were respectively prepared through the same methods by keeping the same amount of CNFs, ZIF-67, and ZIF-67/CNFs composite. All the electrodes were immersed in 0.1 M NaOH solution for 20 min to remove the impurities before experiment. For the electrochemical impedance spectroscopy (EIS) characterization, the CNFs modified GCE, ZIF-67 modified GCE, and ZIF-67/CNFs modified GCE were fabricated by dropping 10 µL 1 mg mL^−1^ of CNFs, ZIF-67, and ZIF-67/CNFs suspension in DIW without Nafion on the GCE surface, followed by drying in Ar atmosphere.

## 4. Conclusions

In summary, a ZIF-67/CNFs composite was synthesized by a “one pot” method, and a novel non-enzymatic H_2_O_2_ sensor made of ZIF-67/CNFs has been successfully fabricated. The ZIF-67/CNFs/GCE exhibited enhanced electrocatalytic performance towards H_2_O_2_ in comparison with ZIF-67/GCE due to the accelerated electron transfer rate by CNFs. Besides, the sensor displayed low detection limit, high sensitivity, as well as satisfactory selectivity and long-term stability. Moreover, the sensor was successfully applied in the detection of H_2_O_2_ in milk. This study not only demonstrates the application potential of the ZIF-67/CNFs composite in constructing high-performance non-enzymatic H_2_O_2_ sensor, but also provides an idea for improving the electrocatalytic activity of MOF materials.

## Figures and Tables

**Table 1:** Sensing performance comparison of different non-enzymatic sensor toward H_2_O_2_^1^.

Electrode Description	Detection Limit (µM)	Linear Range (mM)	Sensitivity (µA mM^−1^ cm^−2^)	Reference
Cu–Ni(OH)_2_/GCE	1.5	0.005–0.14	408	[30]
NP-PtNi	1.0	0.01–0.18	-	[31]
Co_3_O_4_-NWs/carbon foam	1.4	0.01–1.4	-	[32]
Pt nanoflower	60	0.1–0.9	104	[33]
Pt-SnO_2_@C	0.1	0.001–0.17	241.1	[34]
ZIF-67/CNFs/GCE	0.62	0.0025–0.19	323	This work

^1^ The dashes in the table represent values that were not reported in the respective references.

**Table 2:** Recovery experiment of detection of H_2_O_2_ in milk samples.

Sample	C_added_ (µM)	C_found_ (µM)	Recovery (%)	RSD (%)
Milk	100	99.5	99.5	1.5
		102.3	102.3	
		99.2	99.2	
		101.6	101.6	
		98.9	98.9	
